# Metabolism of 2-Chloro-4-Nitrophenol in a Gram Negative Bacterium, *Burkholderia* sp. RKJ 800

**DOI:** 10.1371/journal.pone.0038676

**Published:** 2012-06-06

**Authors:** Pankaj Kumar Arora, Rakesh Kumar Jain

**Affiliations:** Environmental Biotechnology, Institute of Microbial Technology (CSIR), Chandigarh, India; University of Minho, Portugal

## Abstract

A 2-Chloro-4-nitrophenol (2C4NP) degrading bacterial strain designated as RKJ 800 was isolated from a pesticide contaminated site of India by enrichment method and utilized 2C4NP as sole source of carbon and energy. The stoichiometric amounts of nitrite and chloride ions were detected during the degradation of 2C4NP. On the basis of thin layer chromatography, high performance liquid chromatography and gas chromatography-mass spectrometry, chlorohydroquinone (CHQ) and hydroquinone (HQ) were identified as major metabolites of the degradation pathway of 2C4NP. Manganese dependent HQ dioxygenase activity was observed in the crude extract of 2C4NP induced cells of the strain RKJ 800 that suggested the cleavage of the HQ to γ-hydroxymuconic semialdehyde. On the basis of the 16S rRNA gene sequencing, strain RKJ 800 was identified as a member of genus *Burkholderia*. Our studies clearly showed that *Burkholderia* sp. RKJ 800 degraded 2-chloro-4-nitrophenol via hydroquinone pathway. The pathway identified in a gram negative bacterium, *Burkholderia* sp. strain RKJ 800 was differed from previously reported 2C4NP degradation pathway in another gram-negative *Burkholderia* sp. SJ98. This is the first report of the formation of CHQ and HQ in the degradation of 2C4NP by any gram-negative bacteria. Laboratory-scale soil microcosm studies showed that strain RKJ 800 is a suitable candidate for bioremediation of 2C4NP contaminated sites.

## Introduction

Chlorinated nitroaromatic compounds (CNAs) are highly toxic chemicals which have many uses in agriculture worldwide, as fungicides, herbicides and pesticides [1], [2]. These chemicals have been classified into three major groups: chloronitrobenzenes, chloronitrophenols, and chloronitrobenzoates. Due to their worldwide uses, CNAs have been released into our environment and create serious problem to the health of humans and animals. The United State Environmental Protection Agency has been listed several CNAs as priority pollutants.

Microbial degradation of CNAs is a complex process compared to the degradation of other xenobiotic compounds. Furthermore, CNAs are considered to be recalcitrant to microbial attack due to electron withdrawing properties of chloro and nitro groups [3]. Many pure cultures of bacteria have been isolated from their ability to utilize CNAs as the sole source of carbon and energy. The basic mechanism of microbial degradation of CNAs involves the removal of the electron withdrawing nitro or chloro groups that is a key step of the degradation of CNAs as it reduces the recalcitrance nature of CNAs to biodegradation. Two different ways have been reported for removal of the nitro groups: (i) oxidative removal of nitro groups in which nitro groups are directly removed as nitrite ions via oxidative hydroxylation, and (ii) partial reduction of nitro groups in which nitro groups are reduced to hydroxylamines or amino groups by reduction mechanism. The partial reduction of nitro groups has been reported in the most cases of microbial degradation of CNAs [4], [5], [6], [7], [8], [9]. However, the oxidative removal of nitro groups has also been reported in the degradation of CNAs [1], [10], [11], [12]. The chloro groups from CNAs may be removed either prior or after the ring cleavage of aromatic ring. Three different mechanisms have involved in the removal of chloro group: reductive, oxygenolytic and hydrolytic dehalogenation [13]. Pandey et al. [14] reported reductive dehalogenation of 2-chloro-4-nitrophenol (2C4NP) by *Burkholderia* sp. SJ98. Oxygenolytic dehalogenation was reported in the degradation of 2-chloro-4-nitrobenzoic acid by *Acinetobacter* sp. RKJ12 [12].

In this communication, we report degradation of 2C4NP by newly isolated bacterium *Burkholderia* sp. RKJ 800. To date, only few bacteria have been isolated and characterized with their ability to utilize 2C4NP as the sole source of carbon and energy. Examples of these bacteria are *Burkholderia* sp. SJ98 [14], *Arthrobacter nitrophenolicus* SJCon [15], and *Rhodococcus imtechensis* RKJ300 [11]. Strain SJ98 initiated degradation of 2C4NP via reductive dehalogenation with formation of 4-nitrophenol (PNP). The enzyme responsible for reductive dehalogenation was identified as reductive dehalogenase [14]. Further degradation of PNP was sequentially catalyzed by PNP-2-monooxygenase, 4-nitrocatechol-4-monooxygenase, and benezenetriol dioxygenase [14]. Another 2C4NP degrading bacterium, *Arthrobacter nitrophenolicus* SJCon degraded 2C4NP with the formation of chlorohydroquinone (CHQ) that further cleaved to maleylacetate by CHQ dioxygenase [1]. *Rhodococcus imtechensis* RKJ300 degraded 2C4NP with formation of hydroquinone (HQ) that was further cleaved into gamma-hydroxymuconic semialdehyde by ferrous dependent HQ dioxygenase [11].

## Materials and Methods

### Isolation of 2-chloro-4-nitrphenol Degrading Bacterium

A 2C4NP mineralizing strain RKJ 800 was isolated from the soil collected from a pesticide contaminated site, India by enrichment method. No specific permits were required for collection the sample from a pesticide contaminated site. No specific permits were required for the described field studies. I confirm that the location is not privately-owned or protected in any way. I confirm that the field studies did not involve endangered or protected species.

For enrichment, 1 g of the soil sample was added to 250 ml Erlenmeyer flask containing 100 ml minimal media and 0.2 mM 2C4NP (a yellow coloured compound) as the sole source of carbon and energy. On the decolourization, culture was serially diluted, plated on 2C4NP agar plates and plates were incubated at room temperature for 2–5 days. One bacterial strain designated as RKJ 800 was selected from 2C4NP agar plates on the basis of decolorization and used for this study.

Strain RKJ 800 was screened to its ability to degrade other nitroaromatic compounds. For screening, strain RKJ 800 was streaked on minimal agar plates containing 0.3 mM test compound as sole source of carbon and energy. Minimal agar plates were prepared by dissolving the following compounds in 100 ml of double distilled water: 0.4 g Na_2_HPO_4_, 0.2 g KH_2_PO_4_, 0.08 g (NH_4_)_2_SO_4_, 0.08 g MgSO_4_.7H_2_O, 0.1 ml trace element solution and 1.8 g agar. The composition of trace element solution was exactly same as described previously [4]. The media was autoclaved at 15 lbs for 20 min. After autoclave, the desired concentration (0.3 mM) of the filter sterilized test compound was added to the media and media was allowed to cool at room temperature and poured into petri plates. PNP, 4-chloro-2-nitrophenol (4C2NP), 3-methyl-4-nitrophenol (3Me4NP), and 2-chloro-5-nitrophenol (2C5NP) were used as test compounds. Decolourization and growth of strain RKJ 800 on minimal agar plates were considered as positive results.

### Identification of Strain RKJ 800

Strain RKJ 800 was identified by the 16S rRNA gene sequencing using universal primers, 27F (5′-AGAGTTTGATCCTGGCTCAG-3′) and 1492R (5′-TACGGYTACCTTGTTACGACTT-3′) by the method as described previously [16]. The PCR amplification reaction mix (25 µl) contained 50–100 ng of genomic DNA, 2.5 µl of 10 X Taq polymerase buffer, 200 µM of each dNTP, 1.0 U of *Taq* DNA polymerase (New England Biolabs, MA, USA), 20 pmol of each primer (BioBasic Inc. Ontario, Canada) and water. Amplification was carried out using a personal thermocycler (Eppendorf, Hamburg, Germany). Amplification program consisted of an initial denaturation at 94°C for 3 min followed by 30 cycles of denaturation at 94°C for 1 min, annealing at 55°C for 1 min, extension at 70°C for 1 min, and final extension at 72°C for 5 min. The amplified PCR product was sequenced using Big Dye terminator cycle sequencing ready reaction kit (Applied Biosystems) by an automated DNA sequencer (ABI 3130 XL Genetic Analyzer; Applied Biosystems). The 16S rRNA gene sequence similarity of strain RKJ 800 was determined by using BLAST.

### Growth and Degradation Studies

Strain RKJ 800 was grown in 1L Erlenmeyer flask containing 300 ml minimal media and 0.3 mM 2C4NP or PNP or 3Me4NP as the sole source of carbon and energy and the samples were collected at regular intervals. The growth of strain RKJ 800 was measured by taking optical density at 600 nm. The depletion of 2C4NP or 4NP or 3Me4NP was monitored by taking the optical density of the supernatant at 420 nm.

### Detection of Nitrite and Chloride Ions

The nitrite and chloride ions were detected spectrophotometrically by colorimetric methods. For nitrite release, 100 µl of reagent A [0.1% (w/v) sulfanilic acid (Merck) in 30% (v/v) acetic acid] was added into the 100 µl of culture supernatant and mixed properly. After 1 min, 100 µl of reagent B [0.1% (w/v) *N*-(1-naphthyl)-ethylenediamine dihydrochloride (Sigma) in 30% (v/v) acetic acid] was added and incubated at room temperature for 5 min. Presence of nitrite ion in the sample was indicated by the appearance of purple colour and quantified by calculating the absorbance at 540 nm. Concentration of nitrite ion present in spent medium was determined with standard calibration curve of NaNO_2._ The chloride ions were analyzed using QuantiChrom^™^ Chloride assay kit (DICL-250) from BioAssay Systems, Hayward, CA.

### Effect of Various Substrate Concentrations on 2C4NP Degradation

To study the effect of initial 2C4NP concentration, strain RKJ 800 was grown on minimal media containing desired concentration of 2C4NP (0.1 mM, 0.2 mM, 0.3 mM, 0.4 mM and 0.5 mM). Samples were collected at regular intervals. Degradation studies were performed as described above.

### Effect of Different Inoculum Sizes on 2C4NP Degradation

Strain RKJ 800 was grown on 250 ml nutrient broth at 30°C under shaking condition. When the culture reached the late logarithmic phase of growth, usually in 20 to 24 h, the cells were harvested by centrifugation at 8000 × g for 30 min at 4°C, washed with fresh salt solution. The resultant pellets were re-suspended in filter-sterile distilled water. To study effect of different inoculum sizes on degradation, different quantities of cells suspension were added to 100 ml minimal media containing 0.3 mM 2C4NP as a sole source of carbon and energy. At different time intervals, the 2C4NP degradation was monitored. The final concentrations of the inoculum used in this study were: 1.8×10^6^, 3×10^6^, and 2×10^8^ CFU/ml. These inoculum sizes were confirmed at the start of the experiment by plate count.

### Identification of Metabolites

Samples collected at different intervals were centrifuged and supernatants were extracted with ethyl acetate. Extracted samples were analyzed by thin layer chromatography (TLC), high performance liquid chromatography (HPLC) and gas chromatography-mass spectrometry (GC-MS).

TLC was performed using pre-coated silica gel 60 F_254_ plates (20×20 cm, 0.25 mm; Merck, Germany) with solvent (toluene: ethyl acetate: glacial acetic acid, 60:30:5). The compounds were visualized after the treatment of iodine vapors and sprayed with Folin-Ciocalteu’s reagent.

HPLC analysis was carried out using a Waters 600 model high performance liquid chromatography (HPLC) equipped with a photodiode array detector system. The compounds were separated on a C_18_ reverse-phase silica column using 1% glacial acetic acid in methanol and 1% glacial acetic acid in HPLC grade water at a ratio of 80∶20 as the mobile phase. Flow rate was 1.0 ml/min; injection volume was 15 µl, and the compounds were detected at 280 nm and 300 nm.

GC-MS analysis was carried out using a GC-MS-QP5000 instrument (Shimadzu, Tokyo, Japan) equipped with quadrupole mass filter and DB-1 capillary column with ionization of 70 eV, scan interval 1.5 s and mass range of 50–550 Da. The column temperature was initially increased from 90°C to 180°C at the rate of 5°C/min and then from 180°C to 280°C at the rate of 10°C/min. The carrier gas (nitrogen) flow rate was 10 ml/min.

### Preparation of Crude Extract

Strain RKJ 800 was grown on 200 ml minimal media, 10 mM sodium succinate and 0.3 mM 2C4NP. Cells of strain RKJ 800 were centrifuged just prior to decolourization and washed twice with phosphate buffer (20 mM, 7.4 pH) and resuspended in the same buffer. The cells were sonicated in a sonicator by twenty 30s burst with intermittent 30s cooling on ice. The cell extracts were centrifuged at 4°C for 15 min to remove cell debris and the supernatant was used for enzyme assay. Protein contents were estimated by Bradford method.

### Enzyme Assays

In order to further strengthen the results of biochemical characterization of the 2C4NP degradation and demonstrate the induction of enzymes involved, different enzymatic assays were performed with the induced cells of strain RKJ 800. Chloronitrophenol-4-monooxygenase (CNP-4-monooxygenase) activity was determined by measuring nitrite released from 2C4NP upon incubation with cell-free lysate. The standard reaction mixture contained 50 mM Tris–Cl (pH 8.0), 0.2 mM NADH, 0.08 mM FAD, 1 mM MgSO_4_, 5 mg of cell-free lysate, and 300°µM 2C4NP in a total reaction volume of 1 ml. Reaction was initiated by the addition of 2C4NP in the reaction mix. The sample (100 µl reaction mixture) was collected after 10 min of incubation and subjected to quantitation of the nitrite release in reaction mixture according to the method described above. The sample was also extracted with ethyl acetate and analyzed by GC-MS for identification of final product.

CHQ dehalogenase activity was determined as the total chloride released at 30°C in a reaction contained 100 mM Tris–Acetate buffer (pH 7.5), 0.2 mM NADPH, 5–10 mg of cell-free lysate, and 200 µM of 4C2AP. The final volume of the reaction mixture was 5 ml. Samples were collected at regular intervals and assayed for chloride ions as described above. Samples were also extracted with equal volume of ethylacetate and extracted samples were analyzed by GC-MS to identify the product of reaction.

The HQ dioxygenase activity was determined spectrophotometrically by monitoring the formation of γ-hydroxymuconic semialdehyde at 320 nm. The reaction mixture contained (in a final volume of 1 ml) 20 mM phosphate buffer, 0.1 mM hydroquinone, 0.1 mM manganese sulphate and 0.5–1.0 mg crude extract of the protein. Samples were taken at regular intervals (0, 2, 4 and 6 min) and UV spectra were recorded.

### Ring Cleavage Inhibition Studies

The ring cleavage inhibition study was performed using iron chelator viz., 2,2′-dipyridyl, which is an inhibitor for the ferrous ions dependent ring cleaving dioxygenases. Strain RKJ 800 was inoculated in 500 ml Erlenmeyer flask containing 100 ml minimal media, 0.3 mM 2C4NP, 10 mM sodium succinate and 1.5 mM 2,2′-dipyridyl. Culture samples were collected at regular intervals, centrifuged and supernatants were extracted with ethyl acetate. The extracted samples were analyzed with HPLC as described above. This experiment was also performed using the cells of *Rhodococcus imtechensis* RKJ300 as control.

### Microcosm Studies

Soil used in microcosm studies was collected from outside of the campus. No specific permits were required for collection the sample for microcosm studies. The soil contained 37% clay, 28% silt, 35% sand, 0.16% organic carbon, 2.5 ppm phosphorus, 120 ppm potassium, 59 ppm nitrogen and had a pH of 8.8. The pH of the soil was adjusted to 7.0.

Microcosms were prepared using 250 ml glass beakers and each backer contained 50 g of soil spiked with 100 ppm 2C4NP. Four types of microcosms were prepared (a) test microcosm with non-sterile soil, (b) test microcosms with sterile soil, (c) control microcosm with sterile soil, and (b) control microcosm with non-sterile soil. Test microcosms with non-sterile and sterile soils were inoculated with pre-grown and 2C4NP induced cells of strain RKJ 800 at ∼2×10^8^ cells colony-forming units (CFUs) g^−1^ soil, whereas the control microcosms with sterile and non sterile soils were left non-bioaugmented. The bioaugmentation was performed by thorough mixing of the pre-grown cells of strain RKJ 800 with the soil samples. All the microcosms were covered with perforated aluminium foil and incubated at 30°C for 10 days. During the incubation period all the microcosms were sprinkled with distilled water at regular intervals to compensate the loss of water via evaporation. Soil samples were removed at regular intervals, and extracted for analysis. For 2C4NP extraction, 1 g of soil sample was suspended in 10 ml 5% NaOH and vortexed thoroughly for 5 minutes at ambient temperature followed by centrifugation at 1,500 rpm for 10 min. The supernatant was extracted with double volume of ethyl acetate at neutral and acidic pH. The aqueous phases from neutral and acidic extractions were pooled, evaporated to dryness under vacuum in a rotavapor (BUCHI, Switzerland), and finally dissolved in 200 µl methanol and analyzed by HPLC.

The various factors such as inoculum size, pH, temperature, substrate concentration, etc., affecting 2C4NP degradation in microcosm were optimized prior to the study. The optimum cell density for rapid depletion 2C4NP was determined by inoculating spiked soil at final concentrations of 2×10^5^, 2×10^6^, 2×10^7^, 2×10^8^ and 2×10^9^ CFU g^−1^ soil. The optimum temperature and pH concentration for degradation of 2C4NP were tested over a range of 10−60°C and pH 1.5−11.5. Optimum concentration of the compound(s) to be spiked in the soil was determined in a range of 50, 70, 100, 140, and 210 ppm using induced and optimized inoculum concentrations.

## Results

### Isolation and Identification of 2C4NP Degrading Strain

A 2C4NP degrading bacterial strain RKJ 800 was isolated from pesticide-contaminated soil by enrichment method. Strain RKJ 800 was identified as a member of the genus *Burkholderia* on the basis of the 16S rRNA gene sequencing. The 16S rRNA gene sequence of strain RKJ 800 was deposited in Genbank under the accession number HM585226.

### Growth and Degradation Studies

**Figure 1:**
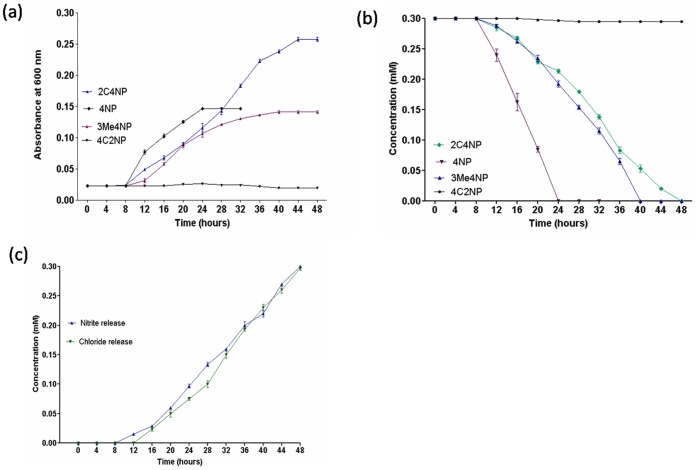
Growth and degradation studies. (a) Utilization of 2C4NP, 4C2NP, PNP and 3Me4NP as sole source of carbon and energy by strain RKJ 800. (b) Degradation of 2C4NP, 4C2NP, PNP and 3Me4NP by strain RKJ 800. (c) Chloride and nitrite releases from 2C4NP by strain RKJ 800.

The degradation and growth studies showed that strain RKJ 800 utilized 2C4NP as sole source of carbon and energy and degraded 2C4NP within 48 h with stoichiometric release of chloride and nitrite ions (Fig. 1a, 1b, 1c). Nitrite release occurred before the chloride release. It indicated that the degradation of 2C4NP was initiated with release of nitrite ions.

*Burkholderia* sp. RKJ 800 was also able to utilize PNP and 3Me4NP as sole source of carbon energy (Fig. 1a, 1b). However, strain RKJ 800 did not utilize 4C2NP and 2C5NP as sole source of carbon and energy.

### Effects of Substrate Concentration on Degradation

**Figure 2:**
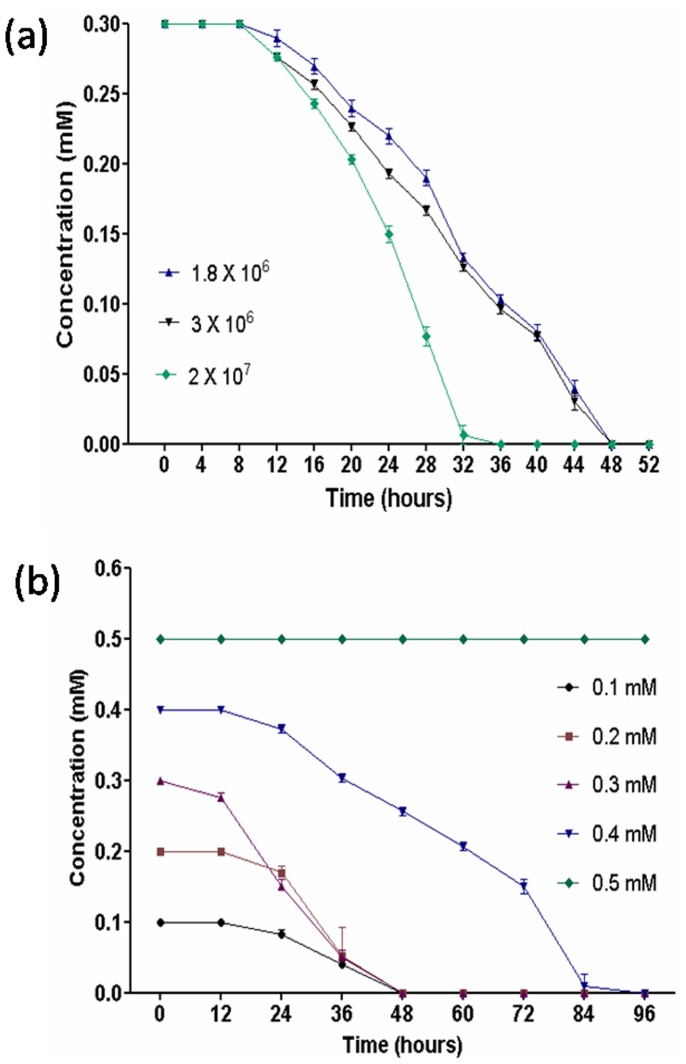
Effect of various substrate concentrations and different inoculum sizes on 2C4NP degradation by strain RKJ 800. (a) Effect of various substrate concentrations. (b) Effect of different inoculum sizes.

No degradation was observed when strain RKJ 800 was grown on minimal medium containing 0.5 mM 2C4NP. Degradation was observed when the range of the 2C4NP concentration was from 0.1 mM to 0.4 mM (Fig. 2a). The optimum concentration for degradation of 2C4NP by strain RKJ 800 was determined as 0.3 mM. This concentration was selected for whole study.

### Effect of Different Inoculum Sizes on 2C4NP Degradation

2C4NP was degraded by strain RKJ 800 during all initial cell densities tested (Fig. 2b). In culture inoculated with highest cell densities, the degradation of 2C4NP occurred rapidly after 16 h and completed within 32 hours. However, in cultures receiving lower inoculum densities, there were progressive decreases of 2C4NP concentration.

### Identification of Metabolites

**Figure 3:**
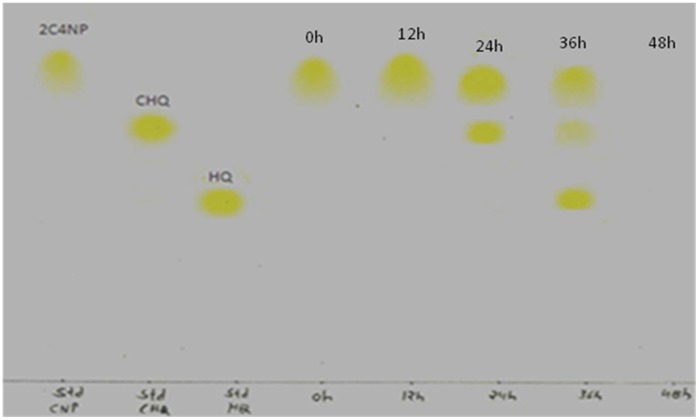
TLC analysis showing the degradation of 2C4NP.

TLC, HPLC and GC-MS studies were carried out to elucidate metabolic pathway of 2C4NP by strain RKJ 800. TLC results indicated depletion of 2C4NP with appearance of the two metabolites (Fig. 3). No metabolite was detected in the sample of 0 h and 12 h. The presence of metabolite I was indicated in the sample of 24 h and 36 h whereas the presence of metabolite II was indicated only in the sample of 36 h. In the sample of 48 h, neither the presence of any metabolite nor parent compound was indicated. The Rf values of metabolite I, II and 2C4NP were 0.64, 0.48 and 0.72 respectively. The Rf values of metabolite I and II exactly matched to that of standard CHQ and HQ respectively. Furthermore, when TLC plates were sprayed with folin ciocalteu’s reagent, an immediate blue coloration was apparent in the case of suspected chlorohydroquinone and hydroquinone.

**Figure 4:**
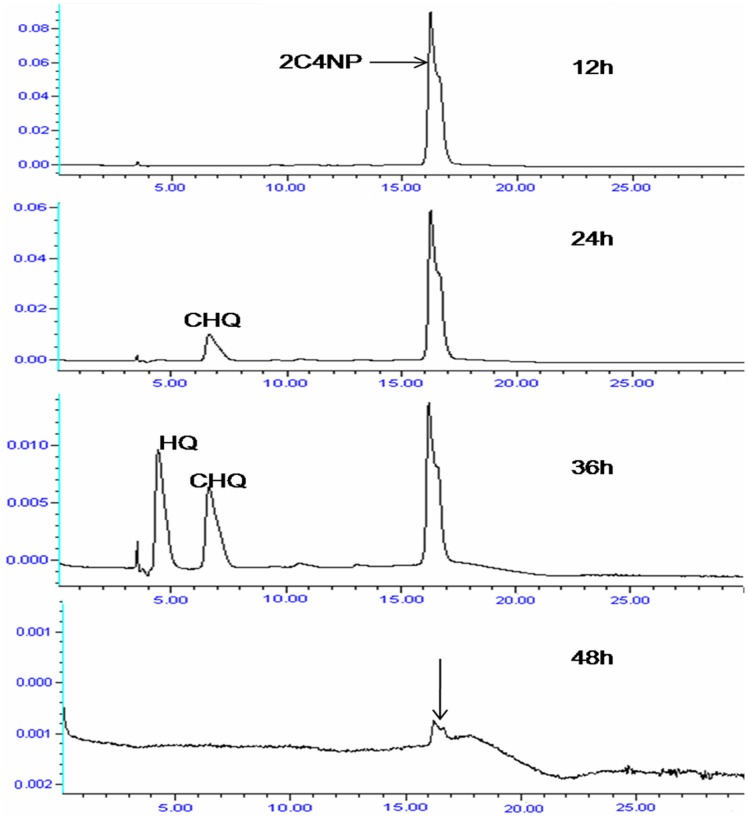
HPLC analysis showing complete depletion of 2C4NP with appearance of metabolites.

HPLC confirmed complete depletion of 2C4NP by strain RKJ 800 within 48 h (Fig. 4). In the 12 h sample, only parent compound was detected. The metabolite I was detected in the sample of 24 h and 36 h whereas the metabolite II was detected only in the 36 h sample. In the sample of 48 h, neither parent compound nor metabolite was detected. The retention time of 2C4NP, metabolite 1 and metabolite II were 16.3 min, 6.7 min and 4.5 min, respectively. The retention times of metabolite I and II were exactly match of authentic CHQ and HQ.

**Figure 5:**
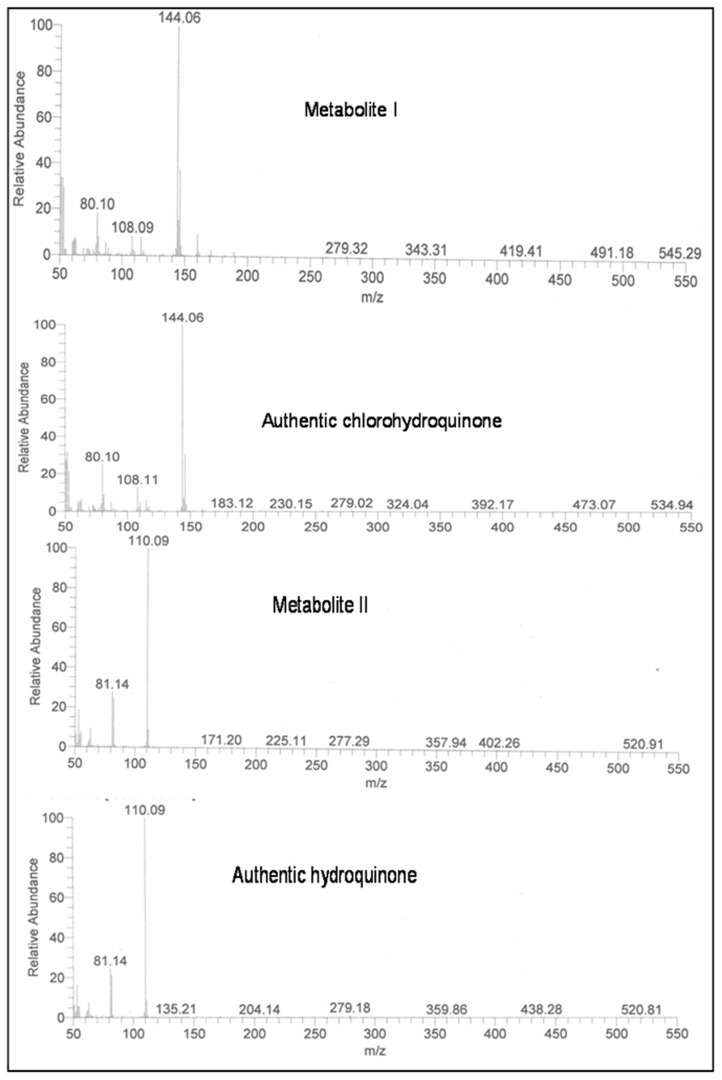
Mass fragment of metabolites I and II with authentic standards.

GC-MS analysis was also carried out to confirm the presence of CHQ and HQ in the degradation pathway of 2C4NP. Mass fragment of 2C4NP was appeared at m/z 173. Mass fragment of the metabolite 1 was observed at m/z 144. This metabolite was formed from 2C4NP by removal of nitro group. The mass fragment of metabolite II was appeared at m/z 110. This metabolite was formed to metabolite I due to reductive removal of chlorine atom. The mass fragment of metabolite I and II exactly matched to that of the authentic CHQ and HQ (Fig. 5).

### Enzyme Assays

In the crude extract of the 2C4NP induced cells of strain RKJ 800, we have detected enzyme activities for CNP-4-monooxygenase, CHQ dehalogenase and HQ dioxygenase. CNP-4-monooxygenase catalyzed the oxidative removal of nitrite ions from 2C4NP. During the enzyme assay, we observed stoichiometric release of nitrite ions (300 µM) that suggested the conversion of 2C4NP to CHQ. Furtheremore, GC-MS analysis of the sample confirmed the formation of CHQ. The mass fragment of product was observed at 144 m/z equivalent to CHQ.

Another enzyme, CHQ dehalogenase catalyzed the conversion of CHQ to HQ with release of chloride ions. The stoichiometric amounts of chloride ions (300 µM) were released during the activity of CHQ dehalogenase that suggested the conversion of CHQ to HQ. The reaction product was identified as HQ on the basis of GC-MS.

**Figure 6:**
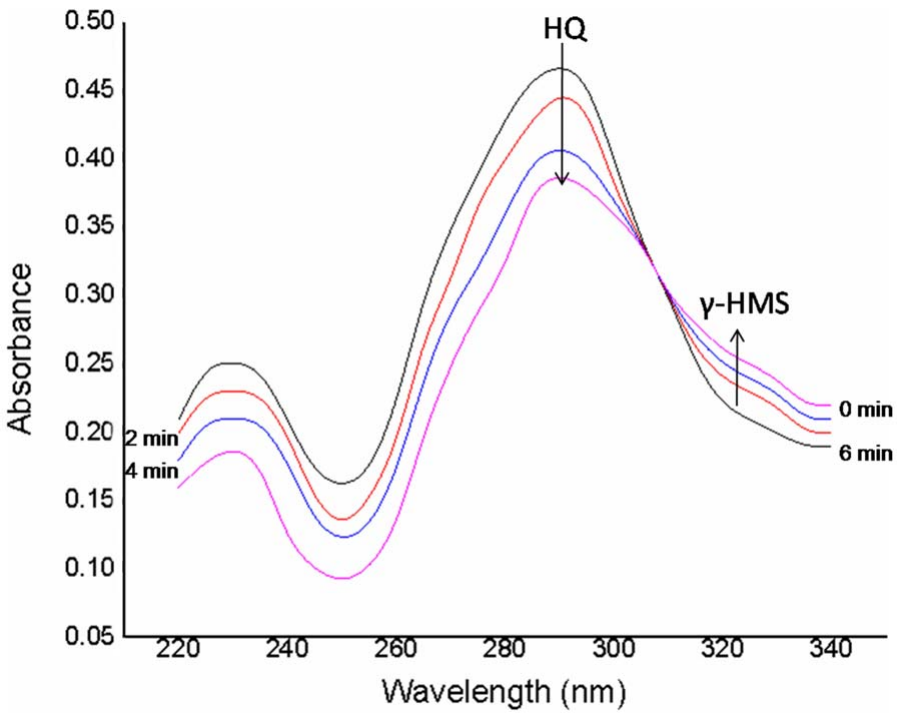
Hydroquinone dioxygenase assay showing depletion of the peak HQ at 289 nm with appearance of the peak of γ-HMS.

The ring cleaving enzyme, HQ dioxygenase catalyzed the conversion of HQ to γ-hydroxymuconic semialdehyde via ring cleavage. The spectrophotometric analysis of HQ dioxygenase assay showed that peak of the HQ at 289 nm was disappeared and peak of γ-hydroxymuconic semialdehyde (HMS) around 320 nm was appeared (Fig. 6).

### Ring Cleavage Inhibition Studies

**Figure 7:**
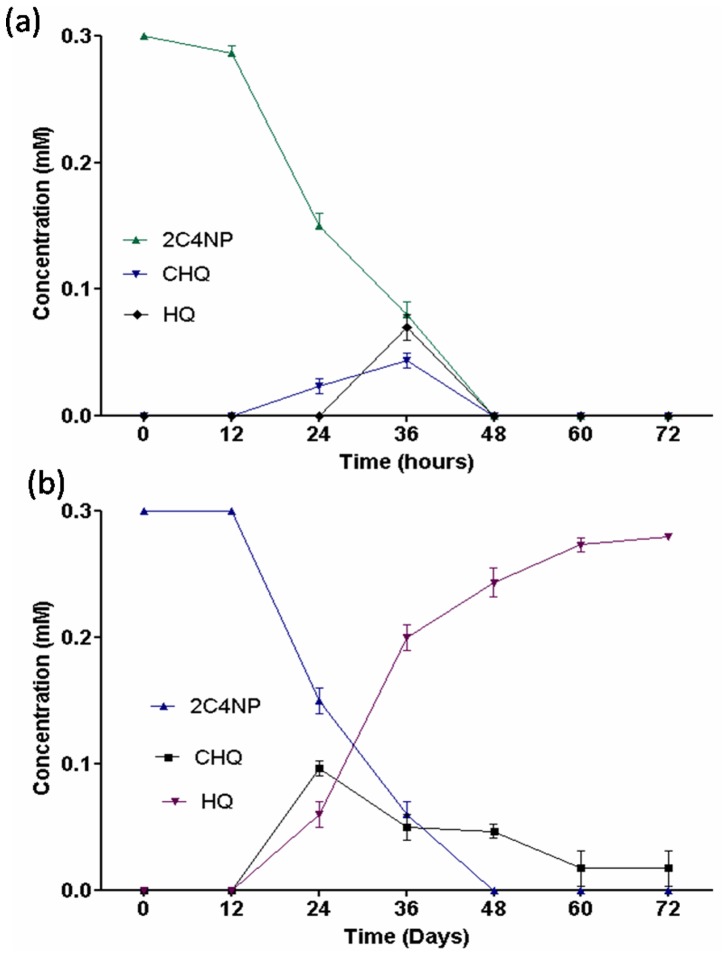
Ring cleavage inhibition studies using 2,2′-dipyridyl . (a) there is no effect on 2,2′-dipyridyl in degradation of 2C4NP by strain RKJ 800. (b) 2,2′-dipyridyl blocks the degradation of HQ in the degradation of 2C4NP by strain RKJ300, therefore, HQ was accumulated.

Generally, ferrous ion is required for enzymatic activity of HQ dioxygenase that cleave HQ to γ-hydroxymuconic semialdehyde [17]. 2,2′-dipyridyl is an inhibitor of ferrous ions which is required for HQ dioxygenase activity [17]. When we have added 2,2′-dipyridyl in the minimal media containing 0.3 mM 2C4NP, 10 mM sodium succinate and 2% inoculums of overnight grown strain RKJ 800, there is no accumulation of HQ suggesting ferrous ions were not involved in HQ dioxygenase activity in strain RKJ 800 (Fig 7a). However, ferrous dependent HQ dioxygenase activity was observed in another 2C4NP degrading bacterium, *Rhodococcus imtechensis* RKJ300 (control cells). When we have added 2, 2′dipyridyl (1.5 mM) in the minimal media containing 0.3 mM 2C4NP, 10 mM sodium succinate and 2% inoculums of overnight grown cells of gram positive bacterial strain RKJ300, 2,2′-dipyridyl chalets the ferrous ions and HQ accumulated in the media (Fig. 7b).

### Microcosm Studies

In order to determine the capability of strain RKJ 800 to degrade 2C4NP in the soil, we performed microcosm studies using both sterile and non-sterile soils under optimized conditions. The optimized parameters were as follows: inoculum size 2×10^8^ CFU g^−1^ soil, pH 7.0, temperature 30°C, and substrate concentration 100 ppm of 2C4NP.

**Figure 8:**
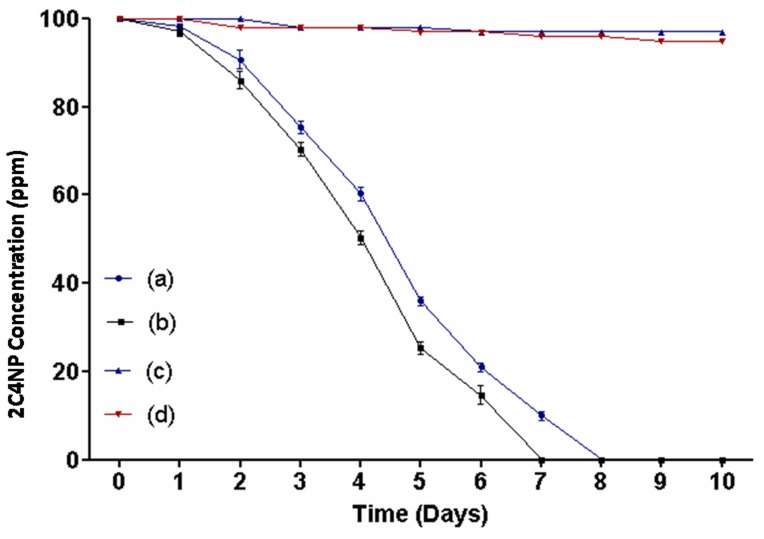
Degradation of 2C4NP by strain RKJ 800 during microcosm studies . (a) Microcosm with sterile soil. (b) Microcosm with non sterile soil. (c) Control with sterile soil.(d) Control with non-sterile soil.

In the test microcosm with sterile soil, there was complete removal of 2C4NP by strain RKJ 800 within 8 days (Fig. 8). There was very slow degradation within initial two days (10%) and after 2 days, degradation was slightly increased and achieved 40% at 4 days. On the sixth days, almost 79% degradation of 2C4NP was completed. The rest 20% degradation was also achieved by 8 days. In the another test microcosm with non-sterile soil, 50 % 2C4NP depletion occurred in the initial 4 days. On the fifth days 72% degradation was completed. Within 7 days 2C4NP was completely degraded by strain RKJ 800. However, in controls with sterile and non sterile soils, very low degradation (only 2–5%) was observed within 10 days.

## Discussion

**Figure 9:**
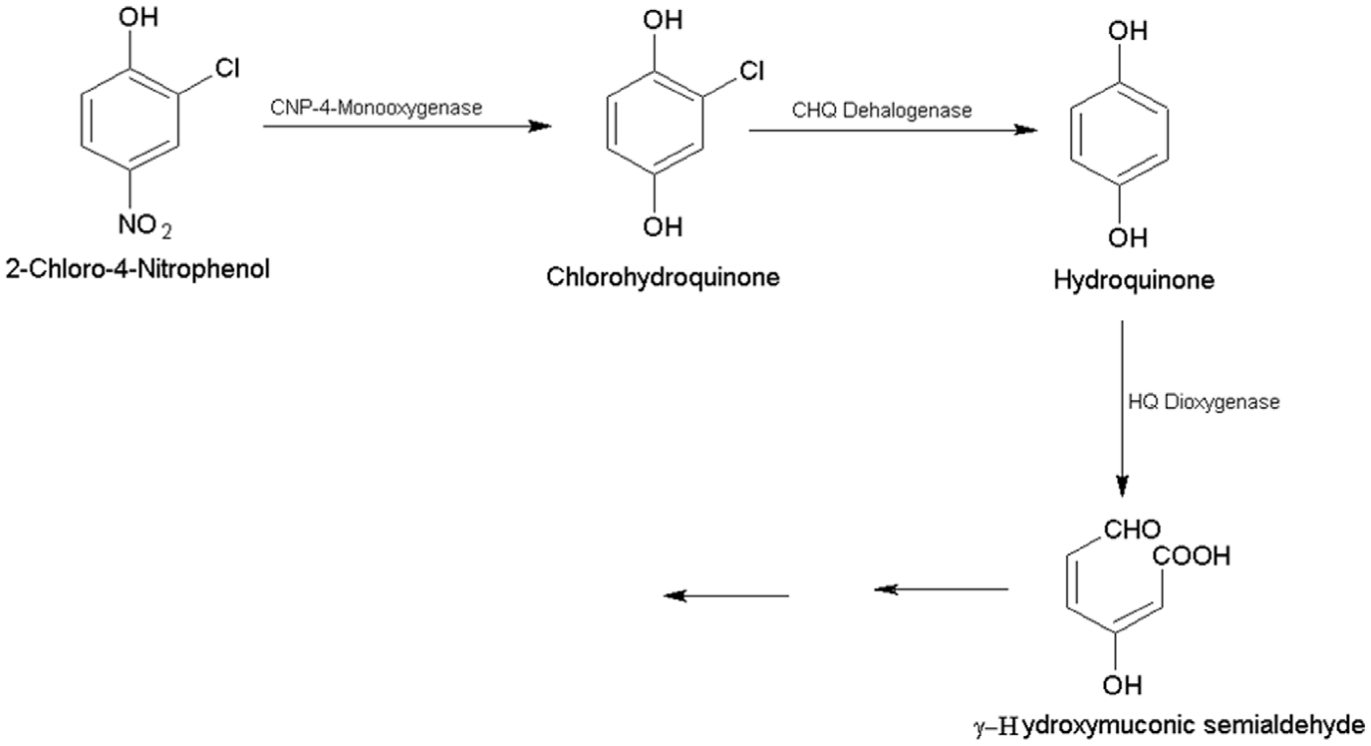
Proposed pathway of degradation of 2C4NP for strain RKJ 800.

*Burkholderia* sp. RKJ 800 utilized 2C4NP as a sole source of carbon and energy and initiated degradation with removal of nitrite ions. CHQ was identified as first metabolite of the 2C4NP degradation that further converted to another metabolite HQ. The degradation of HQ further proceeded via ring cleavage with formation of γ-HMS (Fig. 9).

The degradation pathway identified in *Burkholderia* sp. strain RKJ 800 was differed from the pathway reported in *Burkholderia* sp. SJ98 in which 2C4NP dehalogenated to PNP that was further degraded via formation of nitrocatechol and 1,2,4-benzenetriol [14]. It is very interesting that strain RKJ 800 showed more than 98% 16S rRNA gene sequence similarity with strain SJ98 and both have different metabolic pathway of 2C4NP that indicated involvement of the different enzyme systems in the degradation of 2C4NP in the strains RKJ 800 and SJ98. The enzymes responsible for 2C4NP degradation in strain SJ98 have been identified as reductive dehalogenase, PNP-2-monooxygenase, 4-nitrocatechol-4-monooxygenase, benzenetriol dioxygenase [14]. However, we have detected enzyme activities of CNP-4-monooxygease, CHQ dehalogenase and HQ dioxygenase in the crude extract of 2C4NP induced cells of strain RKJ 800 that suggested involvement of these enzymes in the degradation of 2C4NP by strain RKJ 800.

Recently, we have reported degradation pathway of 2C4NP in *Arthrobacter nitrophenolicus* SJCon in which CHQ was cleaved into maleylacetate [1], [15]. However, in this study, CHQ was dehalogenated to HQ. Dehalogenation of CHQ to HQ was also reported in the degradation pathway of gamma-hexachlorocyclohexane, pentachlorophenol and 2,4,6-trichlorophenol [18], [19], [20].

Ghosh et al. [11] previously reported the presence of CHQ and HQ in the degradation pathway of 2C4NP in gram-positive bacterium *Rhodococcus imtechnesis* RKJ300. It clearly indicated that the metabolic pathway of 2C4NP in gram-negative bacterial strain RKJ 800 is similar to that of found in gram-positive bacterial strain RKJ300. However, there are some interesting differences between in the degradation pathway of 2C4NP in gram-positive bacterium RKJ300 and gram-negative bacterium RKJ 800. We have observed hydroquinone dioxygenase activity in crude extract of 2C4NP induced cells of strain RKJ 800 whereas HQ dioxygenase activity in strain RKJ300 was ferrous ion dependent.

2C4NP is structurally very similar to 4C2NP and 2C5NP. These compounds have same molecular formula (C_6_H_4_NO_3_Cl) and molecular weights (173.5). The difference is only the change in positions of the chloro and nitro groups at benzene rings. Interestingly, strain RKJ 800 degraded 2C4NP, but was not able to degrade 4C2NP and 2C5NP. It is due to the fact that enzymes that act at the *para* positions could not act at the *ortho* or *meta* positions or vice versa [3]. The aromatic compounds which have nitro groups at *ortho* or *meta* position are considered to be more recalcitrant to microbial attack compared to the compounds which have nitro groups at *para* postions [3]. Therefore, 4C2NP and 2C5NP are more recalcitrant than 2C4NP. Few reports are available for complete degradation of 2C5NP and 4C2NP. Schenzle et al. [21] reported degradation of 2C5NP by *Ralstonia* eutropha JMP134 that utilized 2C5NP as a sole source of carbon, nitrogen and energy. The mechanism of degradation of 2C5NP was differed from the degradation of 2C4NP. The degradation of 2C5NP was proceeded with release of ammonia and chloride ions. No ammonia release was observed in degradation of 2C4NP. The degradation of 2C5NP was initiated with reductive mechanism with formation of 2-chloro-5-hydroxylaminophenol. However, the initiation of degradation of 2C4NP in strain RKJ 800 was occurred by oxidative mechanism. The oxidative mechanism of degradation was also observed in mineralization of 4C2NP by a genetically engineered bacterium, *Pseudomonas* sp. NW-31 that initiated degradation of 4C2NP via formation of chlorocatechol with release of nitrite ions and further degradation of chlorocatechol was proceeded with release of chloride ions [10]. However, it is not clear when chloride ions were released in the degradation pathway of 4C2NP by strain NW-31. On the other hand, during the study of degradation of 2C4NP by strain RKJ 800, we observed that the chloride ions were released before the ring cleavage.

Literature studies showed that nitrophenols and their derivatives may be degraded by bacteria via the formation of corresponding HQ. Hayatsu et al. [22] reported degradation of 3Me4NP by a *Burkholderia* sp. strain NF100 and identified methylhydroquinone (MeHQ) as a metabolite of degradation of 3Me4NP. Several bacteria have been isolated and characterized that degrade 4NP via formation of HQ [23], [24]. Strain RKJ 800 also utilized 4NP and 3Me4NP as a sole source of carbon and energy and degraded them with formation of HQ and MeHQ, respectively (data not shown).

Bioremediation of 2C4NP by *Burkholderia* sp. RKJ 800 was investigated using soil microcosms. It was observed that the degradation of 2C4NP in non-sterile soil was slightly faster than sterile soil. There was no accumulation of the any intermediate in the soil during the soil microcosm studies. There was no adverse affect of indigenous bacteria on the degradation of 2C4NP by strain RKJ 800. These results indicated that strain RKJ 800 was suitable for use in the bioremediation of 2C4NP contaminated soils. Previously, Ghosh et al. [11] also reported bioremediation of 2C4NP by a gram positive bacterium, *Rhodococcus imtechensis* RKJ300. However, optimal concentration of 2C4NP that was degraded by strain RKJ300 was 70 ppm and the time taken for degradation was 10 days. *Burkholderia* sp. RKJ 800 degraded 100 ppm within 8 days. It is clear that strain RKJ 800 was better degrader of 2C4NP than strain RKJ300.

### Conclusion

*Burkholderia* sp. RKJ 800 degraded 2C4NP via formation of CHQ and HQ. The ring cleavage of HQ into γ-HMS was catalyzed by a manganese dependent HQ dioxygenase. This is the first report of the formation of CHQ and HQ in the degradation of 2C4NP by any gram negative bacteria. Furthermore, microcosm studies showed that strain RKJ 800 may be used for the bioremediation of 2C4NP contaminated site.

## References

[1] Arora PK, Jain RK (2011). Pathway for degradation of 4-chloro-2-nitrophenol by *Arthrobacter* sp. SJCon.. Curr Microbiol.

[2] Li BZ, Xu XY, Zhu L (2010). Catalytic ozonation-biological coupled processes for the treatment of industrial wastewater containing refractory chlorinated nitroaromatic compounds.. J Zhejiang Univ-Sci B.

[3] Arora PK, Sasikala C, Ramana CV (2012). Degradation of chlorinated nitroaromatic compounds.. Appl Microbiol Biotechnol.

[4] Arora PK, Jain RK (2012). Biotransformation of 4-chloro-2-nitrophenol into 5-chloro-2-methylbenzoxazole by a marine *Bacillus* sp. strain MW-1.. Biodegradation.

[5] Beunink J, Rehm HJ (1990). Coupled reductive and oxidative degradation of 4-chloro-2-nitrophenol by a co-immobilized mixed culture system.. Appl Microbiol Biotechnol.

[6] Katsivela E, Wray V, Pieper DH, Wittich RM (1999). Initial reactions in the biodegradation of 1-chloro-4-nitrobenzene by a newly isolated bacterium, strain LW1.. Appl Environ Microbiol.

[7] Wu HZ, Wei CH, Wang YQ, He QC, Liang SZ (2009). Degradation of o-chloronitrobenzene as the sole carbon and nitrogen sources by *Pseudomonas putida* OCNB-1.. J Environ Sci-China.

[8] Wu JF, Jiang CY, Wang BJ, Ma YF, Liu ZP (2006). Novel partial reductive pathway for 4-chloronitrobenzene and nitrobenzene degradation in *Comamonas* sp. strain CNB-1.. Appl Environ Microbiol.

[9] Zhen D, Liu H, Wang SJ, Zhang JJ, Zhao F (2006). Plasmid-mediated degradation of 4-chloronitrobenzene by newly isolated *Pseudomonas putida* strain ZWL73.. Appl Microbiol Biotechnol.

[10] Bruhn C, Bayly RC, Knackmuss HJ (1988). The Invivo construction of 4-chloro-2-nitrophenol Assimilatory Bacteria.. Arch Microbiol.

[11] Ghosh A, Khurana M, Chauhan A, Takeo M, Chakraborti AK (2010). Degradation of 4-nitrophenol, 2-chloro-4-nitrophenol, and 2,4-dinitrophenol by *Rhodococcus imtechensis* strain RKJ300.. Environ Sci Tech.

[12] Prakash D, Kumar R, Jain RK, Tiwary BN (2011). Novel pathway for the degradation of 2-chloro-4-nitrobenzoic acid by *Acinetobacter* sp. strain RKJ12.. Appl Environ Microbiol.

[13] Arora PK, Srivastava A, Singh VP (2010). Application of monooxygenases in dehalogenation, desulphurization, denitrification and hydroxylation of aromatic compounds.. J Bioremed Biodegrad.

[14] Pandey J, Heipieper HJ, Chauhan A, Arora PK, Prakash D (2011). Reductive dehalogenation mediated initiation of aerobic degradation of 2-chloro-4-nitrophenol (2C4NP) by *Burkholderia* sp. strain SJ98.. Appl Microbiol Biotechnol.

[15] Arora PK, Jain RK (2012). *Arthrobacter nitrophenolicus* sp.. http://dx.doi.org/10.1007/s13205-012-0066-4.

[16] Arora PK, Chauhan A, Pant B, Korpole S, Mayilraj S (2011). *Chryseomicrobium imtechense* gen. nov., sp. nov., a new member of the family Planococcaceae.. Int J Sys Evol Microbiol.

[17] Kolvenbach BA, Lenz M, Benndorf D, Rapp E, Fousek J (2011). Purification and characterization of hydroquinone dioxygenase from *Sphingomonas* sp. strain TTNP3.. AMB Express.

[18] Miyauchi K, Suh SK, Nagata Y, Takagi M (1998). Cloning and sequencing of a 2,5-dichlorohydroquinone reductive dehalogenase gene whose product is involved in degradation of gamma-hexachlorocyclohexane by *Sphingomonas paucimobilis*.. J Bacteriol.

[19] Ohtsubo Y, Miyauchi K, Kanda K, Hatta T, Kiyohara H (1999). PcpA, which is involved in the degradation of pentachlorophenol in *Sphingomonas chlorophenolica* ATCC39723, is a novel type of ring-cleavage dioxygenase.. FEBS Lett.

[20] Reddy GV, Gelpke MD, Gold MH (1998). Degradation of 2,4,6-trichlorophenol by *Phanerochaete chrysosporium*: involvement of reductive dechlorination.. J Bacteriol.

[21] Schenzle A, Lenke H, Spain JC, Knackmuss HJ (1999). Chemoselective nitro group reduction and reductive dechlorination initiate degradation of 2-chloro-5-nitrophenol by *Ralstonia eutropha* JMP134.. Appl Environ Microbiol.

[22] Hayatsu M, Hirano M, Tokuda S (2000). Involvement of two plasmids in fenitrothion degradation by *Burkholderia* sp. strain NF100.. Appl Environ Microbiol.

[23] Spain, JC, Gibson DT (1991). Pathway for biodegradation of *p*-nitrophenol in a *Moraxella* sp. Appl. Environ.. Microbiol.

[24] Ju KS, Parales RE (2010). Nitroaromatic compounds, from synthesis to biodegradation.. Microbiol Mol Biol Rev.

